# In Vitro Glioblastoma Models: A Journey into the Third Dimension

**DOI:** 10.3390/cancers13102449

**Published:** 2021-05-18

**Authors:** Mayra Paolillo, Sergio Comincini, Sergio Schinelli

**Affiliations:** 1 Department of Drug Sciences, University of Pavia, 27100 Pavia, Italy; sergio.schinelli@unipv.it; 2 Department of Biology and Biotechnology “Lazzaro Spallanzani”, University of Pavia, 27100 Pavia, Italy; sergio.comincini@unipv.it

**Keywords:** 3D cell cultures, scaffolds, organoids, organotypic slices, bioprinting

## Abstract

**Simple Summary:**

In this review, the thorny issue of glioblastoma models is addressed, with a focus on 3D in vitro models. In the first part of the manuscript, glioblastoma features and classification are recapitulated, in order to highlight the major critical aspects that should be taken into account when choosing a glioblastoma 3D model. In the second part of the review, the 3D models described in the literature are critically discussed, considering the advantages, disadvantages, and feasibility for each experimental model, in the light of the potential issues that researchers want to address.

**Abstract:**

Glioblastoma multiforme (GBM) is the most lethal primary brain tumor in adults, with an average survival time of about one year from initial diagnosis. In the attempt to overcome the complexity and drawbacks associated with in vivo GBM models, together with the need of developing systems dedicated to screen new potential drugs, considerable efforts have been devoted to the implementation of reliable and affordable in vitro GBM models. Recent findings on GBM molecular features, revealing a high heterogeneity between GBM cells and also between other non-tumor cells belonging to the tumoral niche, have stressed the limitations of the classical 2D cell culture systems. Recently, several novel and innovative 3D cell cultures models for GBM have been proposed and implemented. In this review, we first describe the different populations and their functional role of GBM and niche non-tumor cells that could be used in 3D models. An overview of the current available 3D in vitro systems for modeling GBM, together with their major weaknesses and strengths, is presented. Lastly, we discuss the impact of groundbreaking technologies, such as bioprinting and multi-omics single cell analysis, on the future implementation of 3D in vitro GBM models.

## 1. Introduction

Gliomas are a heterogeneous group of brain tumors among which the higher-grade tumors are glioblastoma multiforme (GBM), that represent the most aggressive and frequent glioma subtype. A common feature of all grades of glioma is their extensive infiltration in the CNS parenchyma [1], which makes surgery for diffuse gliomas unfeasible, since a complete resection can hardly be achieved. Without surgical treatment, survival of GBM patients is less than 12 months from the time of diagnosis and even with current standard treatment, using a combination of surgery, radio-, and classical chemotherapy with the DNA-alkylating agent temozolomide, life expectancy still remains dramatically poor [2]. Unlike for other tumor types, very limited progress in the therapy of high-grade gliomas have been made. Two key factors account for this failure: the highly proliferative and infiltrative behavior of GBM, which prevents the tumor eradication by surgery, thus making classical therapeutic approaches nearly ineffective, and, in addition, the extremely intra- and inter-heterogeneity of tumor mass that make the identification of therapeutic targets very complicated. Many putative genetic and epigenetic drivers of glioma have now been uncovered through systematic genome-wide molecular fingerprinting, opening up a wealth of new directions for drug discovery and more accurate molecular classifications [3,4]. It is expected that this fundamental knowledge will ultimately lead to new treatments and enhanced patient outcomes; however, in the shorter term, there remains an urgent need to identify key aberrant molecular pathways to be targeted by new potential lead compounds in the pharmacological treatment of GBM. However, a critical factor that heavily hampers progress in GBM treatment is the lack of suitable and reliable in vitro models that should drive the choice of subsequent more comprehensive and sophisticated in vivo GBM animal models. In standard 2D cultures, cells are layered on a highly rigid plastic substrate, and then maintained in a medium supplemented with extracellular matrix (ECM) proteins. In a slightly similar model, sometimes defined as 2.5D culture or drip culture, cells are directly deposited on a preformed layer of ECM mixture or laminin coated dishes until they grow to form a confluent monolayer. This platform is very suitable for monitoring cell morphology, different types of imaging, and antibody staining and to run functional studies using commercially available and dedicated assay kits [5,6]. Current classical 2D cellular preclinical in vitro models are of limited utility and poorly informative because of several intrinsic limitations that deeply affect the phenotype, cell signaling, and drug response. Critical parameter involved in these cellular effects include inappropriate cell density, gradients of medium components, unphysiological oxygen levels, disruption of original spatial context, lack of interactions with ECM, and other non-tumor cells present in the GBM microenvironment. Hence, the development of more reliable and feasible novel in vitro models appears to be mandatory to get further insights into GBM molecular biology and therapy. Another aspect to take into account when considering GBM and, more generally, brain models is the presence of immune system components. New trends in immunotherapies have greatly extended the armamentarium of therapeutic tools in a vast majority of solid cancers, but this progress in the treatment of GBM have been hampered by the complexity and the relatively poor knowledge either of the tumor microenvironment in GBM and of the interactions among cancer cells and other immune system components in the brain [7]. Despite these problems, attempts to involve the putative therapeutic actions of the immune system in counteracting GBM malignancy, including approaches such as immune checkpoint modulators, cancer vaccines, and CarT-cell therapies, are currently actively exploited. However, the translation of these therapeutic tools into reliable in vitro models poses several critical challenges. In particular, several works carried out in vivo using animal models have shown that tumor associated macrophages (TAM) could play a crucial and not yet well-defined role in modulating the immune response in GBM microenvironment [8]. Doxorubicin-loaded tumor associated macrophages (TAM) were found to infiltrate GBM spheroids, thus releasing their drug cargo [9] while drug-damaged GBM spheroids attract and reprogram TAMs inducing an anti-GBM phenotype. This interesting finding strongly suggests that the development and refinement of innovative in vitro coculture GBM models are needed to understand the cross-talk between GBM cells and the brain immune system. The development of innovative in vitro 3D tumor models has the potential to fill this gap, thus allowing to improve our knowledge of GBM pathophysiology and to serve as screening tool for new putative molecule susceptible to become anti-GBM drugs. Indeed, recent developments in the implementation of innovative 3D tumor models have revealed that this novel approach could mirror more closely than 2D systems the real in vivo tumor microenvironment. In 3D-embedded culture, cells are first cultured within a gel or liquid mixture of cell-compatible ECM protein, and then maintained in a classical growth medium [10]. This platform can be assembled within microfluidic devices with the great advantages of reducing the amount of precious material, such as rare cells, and increase the sensitivity for analyte measurement or functional assays. In contrast, in mechanically supported 3D models, cells or organ slices and sometimes part of a whole organs are layered or embedded in a solid tissue culture inert surface, with different mechanical properties, that functions as a scaffold, which is then completely submerged in a classical growth medium [11,12]. These two models can incorporate different ECM components, multicellular tissues or different cell types in coculture being ideal to study interplay and crosstalk among tumor cells and microenvironment non-tumor cells.

In the attempt of giving a brief overview of trends in this exciting field, in this review, we will first describe the current classification of GBM subtypes followed by a discussion on GBM heterogeneity. The functional role of non-tumor cells in the tumor niche and in GBM microenvironment, together with their interplay with GBM cells, will be discussed in the attempt to identify essential elements of the tumor niche that should be present in a complex 3D model. Next, we will illustrate recent advancements in the implementation of current 3D in vitro models with an emphasis on their respective advantages and limitations (Table 1). Finally, in the last section, we will briefly discuss innovative single cell multi-omics and bioprinting technologies that in the short future could represent an ideal complementary and ultra-informative approach in shaping and improving the versatility and reliability of 3D GBM in vitro. Readers interested in deepening their knowledge in this field are warmly invited to consult excellent and comprehensive reviews describing not only in details the various features and properties of in vitro and in vivo GBM models, but also the complex platform aimed at studying interaction among different cell types in the GBM niche [5,10,11,12].

## 2. GBM Classification

The GBM heterogeneity is a key issue not only as a basis to understand the physiopathology of GBM, but especially as a starting point to implement and develop reliable in vitro GBM models. This feature has been extensively investigated using a combination of genome-, transcriptomic-, exome-, and epigenome-wide sequencing technologies [70]. Indeed, an enormous wealth of data has led to hypothesize that GBM heterogeneity could be classified according to at least three different levels: (a) genetic and transcriptional level, (b) cellular and tissue level and, finally, (c) tumor location level [71]. Genomic studies have shown that the most relevant changes found in GBM specimens, compared to brain normal tissues, include genomic rearrangement with activating point mutations in EGFR, MET, and PDGFRA receptors, dysregulation of downstream PI3K- and AKT-dependent signaling pathways, loss of tumor suppressor CDKN2A, TP53, RB, PTEN and NF1, and mutations in the TERT promoter [72]. Transcriptomic studies, based on clustering analysis of microarray data reported in The Cancer Genome Atlas (TGCA), have demonstrated that, according to the main pathway found altered in GBM specimens, four main tumor subtypes can be identified: classical, proneural, neural, and mesenchymal [73]. Recently, this former classification has been revisited by other authors who proposed a more restricted classification, encompassing three subtypes instead of four and eliminating the neural subtype although, in certain cases, the reliability of this novel classification remains a matter of debate in the clinical setting. However, it should be pointed out that this classification is an over-simplification that underestimates the dynamic mechanisms of GBM evolution; indeed, transition from a subtype to another due to cancer spontaneous or treatment-induced progression is likely to be more common than previously thought [74]. The majority of GBM cells display a small round shape morphology and are characterized at the genomic level by EGFR amplification, found in almost two/third of the total cell population [16]. These cells, reminiscent of neuronal subtype, often display specific neuronal marker expression such as synaptophysin and MYC amplification. In GBM specimens, multinucleate giant cells and other packed rhomboid-like cells with abundant cytoplasm, probably derived from epithelium, have also been found [17]. The limitations of the classical histopathological diagnosis of GBM tumors, mainly due to broad intracellular variability, could be partially overcome by the aid of DNA methylation-based classification. This approach has been deeply investigated by a report [75] that revealed that this epigenetic information could improve diagnostic precision over standard methods, thus leading to changes of diagnosis in about 12% of examined cases. However, the capability of GBM in vitro models to retain the same pattern of DNA methylation found in the original GBM specimen has been poorly investigated, being limited to GCS models. In one published work, aimed at studying the effects of DNA methylation resetting in GNS cells on malignant behavior, Stricker et al. reported that glioblastoma-derived neural stem cells (GNS) display aberrant specific DNA methylation marks, together with the most common epigenetic anomalies, that are frequently observed in the primary GBM tumors [76]. In another similar study [77], an in-depth analysis of the DNA methylation landscape of GBM-derived cancer stem cells (GSCs) demonstrated that these cells retain the same methylation signature also found in primary GBM-derived xenograft tumors. These two investigations suggest that GCS culture conditions retain most of their original epigenetic pattern, thus supporting and strengthening the notion that GSC represent a valid and suitable in vitro model to decipher the functional role of epigenetic modification on cellular parameters. One important feature of GBM is represented by the isocitrate dehydrogenase (IDH) 1 and 2 status; mutations in IDH genes have been found in the majority of low-grade gliomas and secondary high-grade gliomas. These mutations induce the enzymes to synthesize 2-hydroxyglutarate, a possible oncometabolite, and inhibit NADPH production [78]; de novo GBM (about 90% of diagnosed GBM) are characterized by wild type IDH whilst the remaining 10% that express mutate IDH are supposed to derive from progression of anaplastic or low-grade astrocytoma [79]. Although GBM can arise in virtually any region of the brain, diagnostic analysis by NMR or TAC reveals that about 60% of GBM are localized in frontal, temporal, occipital, and parietal cortex regions, with a minority of cases displaying a multifocal origin [80]. However, this classification has no practical impact in aiding and guiding the therapeutic approach because, at the present time, no difference in cellular heterogeneity has been associated to different GBM localization. A still unsolved issue in this scenario is the finding that brain regions are differentially invaded by GBM; currently, there are no cues (or clues) that could explain, at any investigational level, this differential susceptibility. The genetic diversity is an effective way that GBM cells inside the tumor mass use to develop treatment resistance, a selection process that in turn supports the most typical and deadly factor in GBM malignity: disease recurrence [81]. However, it should be noticed that this classification is no longer in line with recent, dramatic technological advancements, such as the growing field of single cell RNA sequencing, that have introduced an additional layer of complexity demonstrating that GBM tumors actually contain a wide variety of tumor and non-tumor cells of all three subtypes [82]. In terms of tumoral diversity and heterogeneity, the process of culturing GBM cells remains incredibly complicated, because current culture techniques often promote homogeneity in cells, thereby limiting their utility. Different subpopulations of tumor cells have a fundamental role in tumor maintenance and progression. For example, it has recently been shown that differentiated GBM cells communicate bi-directionally with GSCs, promoting GBM progression [83]. Therefore, the selection process currently used to isolate cells, particularly GSCs, from patient samples carries an extremely high risk of losing other tumor subpopulations whose interactions could be critical for tumor behavior. In the light of this evidence, the simplest way to approach this issue relies on the implementation of coculture systems that potentially include one or more nontumor cell populations beyond GBM tumor cells [84].

## 3. Glioma Microenvironment

### 3.1. The Niche

To assure their survival, GBM cells must rely on a complex and dynamic network of communication with other non-tumor cells present in the tumoral niche [71,85,86,87,88]. This favorable environment is created by GBM by inducing phenotype modifications in normal brain tissue cells in order to sustain tumor proliferation and to inhibit the immune system activity towards tumor cells [89]. In the normal human brain, the ratio of astrocyte to neurons is about 10:1, thus stressing the multirole played by astrocytes in regulating almost any process occurring in the brain. Indeed, astrocytes are very abundant in the GBM microenvironment and in the brain tissue surrounding the tumor mass, where they modulate the diffusion of substances, regulate the bioavailability and functionality of neurotransmitters and affect GBM progression by providing and forming peri-vascular spaces necessary for GBM invasiveness and dissemination [90]. Other key players in GBM progression include brain-resident microglia and infiltrating macrophages, especially tumor-associated macrophages (TAMs), representing about 50% of total cells in the tumor mass and promoting GBM growth and invasion [91,92,93,94]. GBM microenvironment also includes T cells that express programmed cell death 1 (PD-1); this finding could partially explain the poor efficacy of checkpoint blockade immunotherapy in glioblastoma [95,96]. In the normal brain microenvironment, neurons stimulate the growth of neural and oligodendrocyte precursor cells, and might play an important role in stem/progenitor cell growth in glioblastoma [83]; however, their participation GBM growth and progression, until very recently, has never been demonstrated or deeply investigated. Venkatesh et al. have demonstrated that the synaptic neuronal protein neuroligin-3 (NLGN3) has a powerful mitogenic activity and soluble NLGN3 was sufficient and necessary to promote high grade glioma cell proliferation in vivo via the PI3K/PTEN/AKT/mTOR signaling pathway [83]. In addition, approximately half of the cells in GBM express genes normally found in postsynaptic elements, and even fewer cells display neuron–glioma synapses [83,97]. These synapses appear to be mainly present on undifferentiated cells, while the bulk of more differentiated tumor cells do not display postsynaptic gene expression [83]. Therefore, the role of neurons in GBM progression remains to be determined and further complicates the picture, increasing the difficulty to set up reliable in vitro models.

### 3.2. ECM

Tumor cells in the brain grow in a complex 3D structure entangled with a brain-specific ECM. For this reason, a key factor that should be considered when implementing in vitro GBM models is the role and composition of ECM, that specifically regulate GBM growth and invasiveness. The ECM can be considered as a physiologic scaffold because of its mechanical properties, but it is also a source of biochemical signals [98] accounting for about 20% of the brain volume with specific composition among different brain regions [99]. During development, cell–ECM interactions regulate cell fate, differentiation, and migration. Similarly, interactions between glioblastoma tumor cells and the ECM play a critical role in invasion and malignancy. Agrin is a large extracellular heparan sulfate proteoglycan and a normal component of the basal lamina of brain vessels; in glioblastoma, agrin content was found to be partially lost from the brain vasculature and replaced by tenascin [100]. Conversely, analyses of protein expression showed that the ECM surrounding the tumor has the same components as healthy brain tissue (collagen IV, fibronectin and laminin), but contains increased levels of hyaluronic acid (HA), as a result of high levels of hyaluronan synthases 1, 2, and 3 (HAS 1/2/3) [101]. Other important ECM-binding proteins and/or modifying enzymes in glioblastoma cells are CD44 (HA receptor), matrix metalloproteinase-9 (MMP9), and hyaluronidases 1/2/3 (Hyal 1/2/3). All these have a direct impact on ECM remodeling, facilitating the invasive and infiltrative phenotype of glioblastoma. In addition, both GBM cells and GBM stem cells have been found to express high levels of RGD-binding integrins [18,102]. These integrins bind to the RGD motif of ECM proteins like laminin, fibronectin, and vitronectin. The binding of integrin receptors to ECM proteins induces important changes in GBM cells and in GBM cancer stem cells, increasing resistance to anoikis and stimulating cell infiltration [103]; integrin receptors have been studied as potential therapeutic targets in GBM and the integrin antagonist Cilengitide gave hopes in high grade glioma therapies, that unfortunately failed in phase III clinical trials [103]. 

## 4. Glioma Cells in 2D and 3D Models

### 4.1. Glioma Cell Lines and GSC

Glioma cell lines are currently used in research because of several advantages, as they are commercially available and less expensive compared to murine models, easy to use, can be propagated in culture virtually indefinitely, and do not pose ethical concerns associated with the use of animal and human tissues. Although glioma cell lines currently represent a reference tool for brain tumor research, this model is flawed by several drawbacks and its use merits critical consideration [13]. First, serial passage of cell lines can cause genotypic and phenotypic variation over an extended period of time, thus complicating the already complicated picture of glioma genotype. In addition, over 20 different culture conditions have been identified in the literature for glioma cell lines: the growth conditions may differ considerably from study to study, making the comparison of studies quite difficult. Glioma have been traditionally cultured in DMEM media supplemented with 10% FBS, which is commonly identified as a differentiating culture condition, while the most common non-differentiating condition is serum-free media, usually supplemented with FGF-2 and EGF [14]. The switch of cell lines from one growth condition to another results in major changes in genotypic and phenotypic cell profiles: serial passages in FBS induces a differentiation toward an astrocytic state that results in genotypic and phenotypic variations accompanied by transcriptional and epigenetic programs that do not mirror the original conditions. In contrast, GBM cells in non-differentiating culture conditions were found to display higher invasive potential, lower drug efflux capacity, and higher sensitivity to immune responses mediated by NK and T-cells [15] when compared to their more differentiated counterparts. This major drawback drastically reduces the utility of these cell lines, clearly indicating that they do not represent a reliable model of human GBM; in the light of these considerations, this cell model appears obsolete and data obtained using cell lines should be interpreted with caution; particularly, this model should not be implemented in 3D models. In the attempt to overcome this gap, neurooncologists have turned their attention to recreate the conditions necessary to maintain in culture GBM cells directly derived from tumor tissue removed during surgical resection. Indeed, groundbreaking works by Galli et al. [19] showed that it is possible to maintain long-term cultures of these GBM-derived cells, termed GSC or TIC, in suspension as neurospheres. GSC owe their name to the fact that they display many of the key features of healthy neural stem cells, including the ability to self-renew, the capacity to generate progeny of a variety of different cell types, and expression of key stem cell-related genes [20,21]. Indeed, the fluctuating gene expression pattern observed in GSC from different sources and under different growth conditions have led scientists to the definition of GSC multipotency as an “emergent property of a cell population in constant dynamic flux” [22]. To achieve GSC growth, the growing medium does not contain FBS that is substituted by specific nutrients mix (N2 and B27) and low amount of epidermal growth factor (EGF) and/or basic fibroblast growth factor (bFGF). Since the first description of cancer stem cells in GBM, a number of papers have accumulated, all clearly indicating that high grade gliomas are driven by cells that display features of neural stem and progenitor cells [14]. GSC were found to share many common features with neural stem cells (NSC); they are capable of indefinite self-renewal and multipotent differentiation, and they express genes that promote NSC phenotypes such as NESTIN and SOX2 [23]. Glioblastoma stem-like cells are highly resistant to therapeutic treatments, radiation, and display high invasiveness of healthy brain [24]. Many of the invasive mechanisms utilized by GSC were found to mimic NSC motility in white matter and blood vessels [16]. Interestingly, GBM tumors display an infiltrative leading edge that disseminates into healthy tissue and orthotopic xenograft tumors derived from human GSC mimic this invasive behavior displaying GSC concentrated at the tumor edge [17]. The reported GSC resistance to radiation and conventional chemotherapeutics, including temozolomide, are currently ascribed to a number of factors: signaling pathways involved in tumor cell contacts and tumor–stroma interactions, including the Wnt/β-catenin, the Notch, PI3K, and JAK/STAT pathways [17]. Moreover, transcription factors have been involved, such as c-Myc, BMI1, SOX2, NANOG, OCT4, and ID1 [78]. Enhanced activity of the DNA damage repair O-6-Methylguanine-DNA methyltransferase (MGMT), a DNA repair enzyme whose expression level is regulated epigenetically at the gene promoter, also correlates with resistance to chemotherapies and renders GSC resistant to temozolomide [2]. Another interesting aspect is the finding that recurrent GBM are enriched in GSC, compared to the primary tumor, suggesting that GSC play a dominant role in GBM relapse [2] and, therefore, are the most reliable target to inhibit relapses. An important feature of GSC is the ability to differentiate into non-tumorigenic cancer-associated cells, such as vascular cells, that contribute to the formation of the tumor niche [25]. This potential ability to differentiate into different cell types is currently considered to contribute to the well-known cellular heterogeneity of GBM, a feature that has been reproduced also by in vivo tumorigenicity experiments [26]. Besides determining the proper culture conditions, purification of GSC requires a multistep process to test self-renewal, multipotency, and stem-marker expression. To identify and enrich GSC cultures by cell sorting is a necessary passage and, therefore, great efforts have been devolved in identifying and validating cell surface biomarkers [27]. However, to definitively confirm GSC nature, functional analysis is mandatory, particularly tumorigenicity tests in vivo. Under these conditions, GSC derived from patient tumor samples represent a very suitable model to test new drugs in vitro [18]. Apart from these considerations, and having in mind that this model is not free of pitfalls and drawbacks, the most important features that have fueled the success of the GSC model relies in the finding, clearly shown by comparative successive studies, that these GSC faithfully recapitulate the features of the original primary tumors [28]. Hence, the GSC culture system is currently widely accepted as the best reliable in vitro model of GBM. The neurosphere culture method was successfully used for enrichment of tumor-initiating cells, as they were originally named, from many different solid tumors and, therefore, was also used to select and propagate in culture brain tumors-derived stem cells. [19]. Soon after the successful attempts to isolate GCS, some limitations in the culture method became evident: the spheres could be composed by non homogeneous cell populations, containing also other precursor cells; short-lived progenitor cells also proliferate in suspension; another limitation is that the spheres’ environment was found to limit stem cell divisions and induce cell death and the cells came into contact with drugs or other agents used during experiments unevenly in the spheres [29]. These drawbacks limited rigorous assessment of stem cell behavior on bulk populations. However, growth in suspension is not a defining feature of stem cells and is not essential for their long-term expansion and, therefore, methods for deriving and expanding human adherent GSC were reported, providing uniform access to growth factors and drugs without altering GSC features [30]. Cells in adherent monolayers display several advantages over neurososphere culture that include an enhanced degree of homogeneity, easiness of imaging analysis, and the possibility to develop a clonal propagation from a single cell [30]. Our own group and others have also reported a much greater success in deriving new GBM cell lines when using adherent culture, with >90% success for IDH wild-type GBM [15,18,31]. The choice of growing GSC in suspension as neurospheres or in adherent conditions still spurs a harsh debate, thus raising controversies among basic neuro-oncologist; nonetheless, quite surprisingly, only one work has investigated possible functional differences between in vitro GSC grown as neurospheres or laminin-adherent cultures [21]. No differences in cell death, growth, proliferation, apoptosis, dilution clonal frequency, differentiation markers expression, and tumorigenicity in vivo was found, thus leading the authors to conclude that both conditions are functionally equivalent. However, quite notably, RNA microarray analysis showed that AdC displayed 23 upregulated genes, including CD44, and 7 downregulated genes compared to neurospheres, although some of these changes were not statistically significant. Unfortunately, in the paper, the identity of these up- and downregulated genes was not mentioned, thus avoiding a deeper interpretation of the possible role of these mis-regulated genes.

#### 4.1.1. The EGFR Issue

Although the GSC model, irrespective of its culture conditions, displays several advantages compared to classical GBM cell lines, it suffers from one important drawback related to the loss of specific key point mutations, a scenario that is critically epitomized by the constitutively active EGFRvIII receptor and/or EGFR amplification issues [104]. In approximately 50% of GBM specimens, an EGFR amplification can be found, and about 50% of specimens also express the EGFRvIII mutation; these two EGFR modifications greatly amplify EGFR activity which, in turn, impinges on several signaling-dependent pathways regulating key cellular prooncogenic effects such as proliferation, apoptosis, differentiation, and invasiveness. Quite disappointingly, when GSC are cultured in classical maintenance standard medium (containing FGF and EGF), they completely lose EGFRvIII and EGFR amplification, thus inducing a reconsideration of the utility of the GSC model as a tool to study the molecular events underlying GBM malignity and as tool to screen potential new antioncogenic drugs. To bypass this drawback, several authors have tried to preserve, or at least minimize the loss of, EGFR amplification and EGFRvIII by cultivating GSC in the absence of EGF or in subminimal amounts of these growth factors [105]. Although this approach demonstrates that GSC could at least partially retain their genetic heterogeneity, characterized by EGFR amplification/EGFRvIII status, simply by modulating their exposure to different EGF concentrations, this model displays limitations. Indeed, the success of this strategy appears to strictly depend on the initial very high expression of EGFRVIII/EGFR amplification that self-sustains cell survival by an autocrine loop; other pitfalls include the lack of standardized procedures and the possibility that the absence of exogenous EGF could affect GSC survival and maintenance over the long-term period [106]. In conclusion, current culture systems appear to be over-simplified and introduce variables not encountered in the brain. Oxygen concentrations are held constant at 20% by most incubators, while media contain an elevated amount of glucose. Plastic flasks introduce an unnaturally stiff surface, which GBM cells are known to react to. Furthermore, coating flasks with Matrigel, while better than culturing directly on plastic, exposes tumor cells to elevated levels of collagen and laminin. Mono-cultures mean that in vitro experiments fail to account for non-tumor cells’ contributions to the microenvironment [107]. Obviously, this complex structure cannot be reproduced in vitro, but some parameters can be calibrated to resemble more closely the in vivo conditions: the substrate where cells adhere to, nutrients, growth factors, and oxygen levels. Glioma cells grown in spheres represent a sort of 3D model, even though, in this model, an important parameter is lacking, which is the adhesion to a substrate. However, GBM tumor cells in 3D cultures have been found to behave in a different way from cells in classical 2D conditions [32,33], supporting the notion that 3D conditions can better mimic what happens in vivo. Unfortunately, results that partially contradict this assumption were found in another study [34]: 68 patient-derived GBM spheres were analyzed together with the tumors of origin and a very limited overlapping of gene expression patterns was found.

#### 4.1.2. Microtubes

Apart from the EGFR issue, recent in vivo and in vitro findings seem to suggest that GSC grown in adherent conditions or in 3D could better resemble the structure and original architecture of GBM in vivo. Indeed, tumor cells in GBM extend long membrane-like protrusions, called tumor microtubes (TM), that support cell invasion and proliferation, thus allowing long distance communications [35,36]. The TM found in GBM are very similar to their physiological counterpart named tunneling nanotubes (TNTs), acting as a syncytium that allows the flow of ions and other molecules between GBM cells [37,38]. In detail, TNTs are described as thin membranous channels located between cells that can transfer cellular organelles, surface receptors, GPI-anchored proteins, and calcium fluxes. TNTs have been found to develop from injured cells toward healthy cells and are used to transfer cellular contents to healthy cells. In a similar way, TNT and TM have been identified in gliomas, where they have key functions in maintaining intercellular communication between glioma subpopulations. However, glioma cells show a different behavior regarding TM formation and glioma cell subpopulations, with a different invasive behavior, and display TM network with different features. To further complicate the picture, in a recent work, TM formation was shown to occur in astrocytes after 48 h of co-culture with GBM cells in 3D hydrogel models, forming connections between astrocytes and tumor cells and leading to mitochondrial exchange between these cell types [39]. The clinical relevance of these TM is further highlighted by their role resistance to radiotherapy and chemotherapy [40,41,42]. The plethora of mechanisms elicited by TM makes this structure a very attractive and a suitable target for the development of therapies focused on limiting GBM progression and resistance to treatments. Considering these interesting studies, the possibility to develop 3D models where glioma cells can give rise to TM networks completely changes the perspective of 3D models and makes classical 2D cell culture appear a very approximate model. To this end, the possibility to reproduce TM in vitro by improving existing ones or implementing new 3D configurations could represent an invaluable opportunity to raise the status of 3D models from a limited reproducible system to a reliable in vitro gold standard preclinical model.

### 4.2. hiPSC

In the attempt to develop reliable GBM models, scientists take inspiration from the technology used to obtain live normal brain cell in vitro [43]; an interesting approach to investigate human gliomagenesis requires the use of primary embryonic/fetal NPCs that could be then differentiated in GSC-like cells [44], but the ethical controversies surrounding the use of embryonic/fetal material represent a strong limitation to study the role of different mutations associated with malignancy in the context of the human genetic background. These limitations stimulated a novel approach, aimed at obtaining cells with a GSC-like phenotype, based on the reprogramming of human-induced pluripotent stem cells (hiPSC) [45]. This innovative work relies on the genetic manipulation of some signaling pathways in hiPSC, such as p53 and receptor tyrosine kinase-dependent signaling, to obtain hiPSC-derived neural progenitor cells (iNSC) cells that acquire a GSC-like phenotype display typical of GSC-like features such as self-renewal, migratory, and metabolic properties. Notably, iNSCs orthotopically transplanted into murine brain give rise to GBM-like tumors containing undifferentiated stem cells and differentiated derivatives and, in addition, when employed in an anti-GBM agents screening assay, iNSC appeared to be sensitive to three molecules active against specific targets that affect the malignity of GSCs-derived patients [46]. This provocative and intriguing study strongly suggests that a new source of GBM-like cells could be used to develop 3D glioma models, although the validation of this approach requires further studies and experiments aimed at evaluating, at the transcriptomic, genomic, and functional level, similarities and differences between of iNPC cells and GSC directly obtained from primary tumor material.

## 5. Multicellular 3D Tumor Models: Cocultures, Spheroids, and Scaffolds

### 5.1. Cocultures

Another complementary exploited approach, mainly aimed at recreating the GBM tumor niche, is represented by coculture systems. In an interesting study, a 2D co-culture model of microglial (MG) and GBM cells was established and the presence of MG was found to induce drug resistance; however, this protective effect was enhanced when the same cells were grown in 3D [108]. Another elegant demonstration of the reliability of these systems has been successfully recently described [109]; the authors set up a model derived from a spheroid tissue microarray (microTMA) technology, designed for multiplex staining and high-throughput histology analysis of spheroids containing iPSC-derived different neuronal phenotypes, astrocytes, and oligodendrocytes. The authors found that treatments of this platform with anticancer agents decreased tumor size but, more notably, spared the population of non-tumor cells. These data led the authors to propose this platform as an innovative approach to screen new potential anticancer compounds and to evaluate options in personalized treatment. In pursuing the same goals and trying to overcome the pitfalls associated with standard microwell arrays, such as cost and complexity, other authors reported the implementation of self-filling microwell arrays (SFMAs) [110]. These arrays, fabricated by replica of 3D agarose molds and filled with glioma U-87 MG cells, demonstrated their validity in performing drug toxicity and efficacy studies. Although it is still unknown whether this model could be applied to other GBM-like cells, we agree with the authors that their novel approach, maybe further refined and improved, could really open new avenues in development of future 3D models. Co-cultures offer an exciting opportunity to create novel 3D tissue-like models in vitro. The technologies developed for 3D culture can be customized for alternative applications containing different cell types; cells of different types can be co-seeded in suspended hanging drops to create micro-tissue aggregates composed of more than one cell type. Alternatively, when seeded in hydrogel, cells of different types can self-organize and form tissue-like structures as they migrate within the gel. Similarly, a mixture of different cells can be added directly to a porous scaffold where they self-arrange within the matrix.

### 5.2. Spheroids

One approach that has been extensively investigated with the goal to bypass the drawbacks associated with 2D models deals with the formation of 3D spheroids. Basically, spheroids are formed by heterogeneous aggregate of different cell types not attached to any solid surface for support, allowing cells to grow and proliferate freely, thus forming floating cell clusters with spheroid or spheroid-like architecture. This in vitro model has advantages compared to 2D cultures: spheroids maintain the gene expression and genomic pattern almost identical to the original tumor from which they derive; when spheroids are dissociated in single cells, these cells proliferate and rapidly form secondary spheroids that, once implanted in animals, still lead to the formation of GBM-like tumors; the 3D architecture allows the study of the reciprocal relationship among GSC and other cells belonging to the GBM microenvironment such as stromal, immune, and endothelial cells in a coculture system, thus mimicking more closely a physiological context in comparison to other in vitro models. The spheroid model is currently considered to be the most accurate in vitro GBM model, sometimes referred to as the gold standard in the field [111]. The major advantage of this model, compared to other in vitro GBM models, resides in the possibility to maintain spheroids for a long term, thus allowing the long-term and high-throughput monitoring of chronic treatments with agents or drugs on cellular parameters [112]. In addition, spheroids may represent a model to study cell-to-cell interactions both in the outer layer and in an internal core, characterized by hypoxic and necrotic regions that resemble a common feature of GBM tumor mass in situ. However, it is important to underline that spheroids have some crucial disadvantages that somehow could severely limit their broad use: usually, unless cells are cocultured as discussed above, spheroids lack other microenvironment non-tumor cells that are well known to affect their features; although a common belief states that GSC spheroids are constituted by an homogeneous cell populations, certain data obtained in cell limiting dilution assay showed that this assertion is likely false, thus requiring additional experiments by single cell technologies; finally, the spheroid conformation elicits an outer–inner layer-dependent decreasing gradient of concentration of nutrients, agents, and drugs with outer cells more exposed to agents in comparison to the internal core [113]. These considerations suggest that several precautions, such as avoiding the formation of excessive large spheroids, the use of a homogeneous size of spheroids, and the standardization of initial clonal dilutions should be carefully evaluated to get the most from a balanced compromise between relative advantages and disadvantages of this model. The reliability of GSC spheroids as in vitro model has also been severely questioned by the finding that drugs, such as erlotinib, that demonstrated good efficacy in GSC spheroid model failed when tested in clinical trials [114,115]. In a recent study [116], the authors took an innovative approach to get closer to the therapeutic goal of precision oncology. The authors derived a large number of tumor-sphere-forming patient-derived cell cultures (PDCs), obtained from surgical specimens cultured in serum-free medium. After six days in culture, the authors showed that the PDCs retained the molecular characteristics, such as somatic variants and copy number alterations, of their parental tumor tissues. These PDCs, derived from hundreds of patients with different cancer types, were then used to successfully screen 60 anticancer drugs. Perhaps the most striking finding, especially in the GBM modelling context, was that glioma PDCs, unlike the majority of 2D systems, retain genomic alterations in EGFR such as amplification, mutation, or deletion of exons 2–7 (EGFRvIII). This important feature of PDCs allowed the authors to pinpoint a crucial link between expression of NRG1, a ligand for EGFR family members, together with upregulation of the PI3K–AKT–mTOR pathway, and resistance to EGFR inhibitors.

### 5.3. Scaffolds

**Figure 1:**
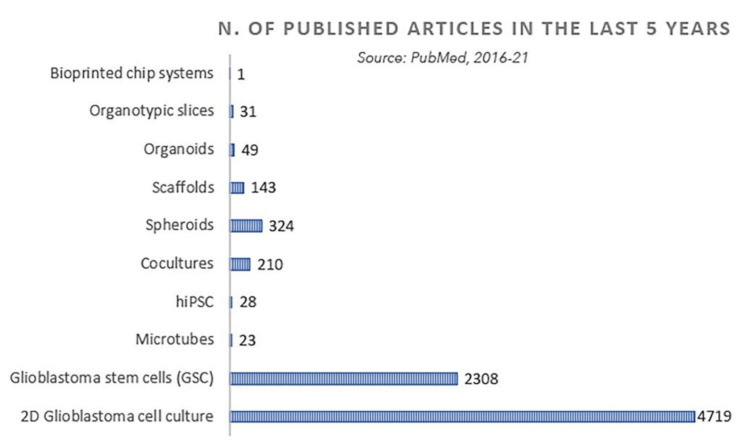
Number of published papers using or referring to the GBM experimental models reviewed above. Source: PubMed.

In addition to spheres, other 3D models have been tested using scaffolds made of different materials, classically divided in rigid and non-rigid scaffolds (Figure 1). One of the most critical drawback of classical 3D model is the lack of an appropriate solid substrate, physiologically represented by ECM, to initially allow the adhesion and subsequently growth and maintenance of GBM cells. Several research teams have therefore devoted their efforts to develop a strategy that exploits the deposition of different types and sometimes combinations of various cell types GBM cells over 3D scaffold structures. The purpose of fabricating scaffolds is to produce tissue-like materials such that they can eventually perform like the native tissues and the idea of scaffold fabrication is based on creating materials with optimum pore size, structure, and porosity for various applications. Generally, scaffolds are first created and cells are subsequently cultured on these scaffolds. As such, there are some existing limitations to this current approach. These prefabricated scaffolds have a certain material property that may or may not be suitable to support normal cell growth or differentiation. Incompatibility of the scaffold with the cellular application will eventually lead to the failure of the entire tissue-engineered scaffolding system. Hence, an in-depth analysis is necessary to evaluate the exact porosity and the pore size that is optimal for each scaffold system such that they complement the intended type of tissue engineering application. In addition, to mimic the actual situation where the ECM undergoes continuous remodeling or healing processes, scaffolds with post-manufacturing tunability are essential in order to provide a suitable microenvironment for these dynamic changes. However, synthetic materials may lack sites for cellular adhesion and may require a coating of ECM proteins to attempt to mimic the niche in which cells reside naturally. There are several different methods for scaffold-based 3D culture, which can be broadly divided into two approaches—hydrogels and solid scaffolds. Here, we will briefly discuss non-rigid systems that have been implemented GBM-like cells or cocultures systems with non-tumor cells belonging to the tumor niche. In particular, glioma cell plasticity has been studied using chitosan-based scaffolds. U118 glioma cells grown on a chitosan scaffold, for example, were reported to form spheres; under these conditions, increased expression of stem markers (Nestin, Musashi-1, and CD44 and increased invasive capacity compared with traditional 2D cultures, were observed [117]. The cells grown under these conditions also displayed increased resistance to both TMZ and doxorubicin, coupled with increased expression of the ABCG2 drug efflux pump, suggesting a phenotypic switch toward a more GSC-like state; similar results were obtained with U87 MG [118].

#### 5.3.1. Hydrogels

Another approach to overcome the lack of GBM microenvironment cells, typical of spheroids, relies on the use of hydrogel scaffolds. Hydrogel are formed by soft water-swollen materials usually composed of natural or synthetic cross-linked polymer chains that can be supplemented with other components, such as ECM proteins, in order to resemble the actual GBM microenvironment. The ECM components, for example, collagen I and IV, laminin, hyaluronic acid, and fibrin, increase the intrinsic biocompatibility of natural of hydrogels and support cell survival and development. Indeed, the presence of these components is crucial for the modulation of a wide variety of cellular effects that include cell proliferation and migration, sensitivity to chemotherapeutics, and cell differentiation [119]. Among non-rigid scaffold, poly(ethylene-glycol) (PEG)-based hydrogels display several advantages; they have good versatility toward mechanic and physical properties and the composition of hydrogels could modulate matrix stiffness without changing the biochemical contents. Wang et al. [120] developed a bioengineered 3D brain tumor PEG-based model, that incorporate hyaluronic acid (HA) and some MMP-cleavable peptides. The authors demonstrated that this system supports U87 GBM cell growth and migration in 3D over several weeks in culture and upregulates ECM remodeling genes. Another aspect to be considered is that GBM cell proliferation, morphology, migration, and ECM deposition are strictly dependent of variations on matrix stiffness, which is regulated by HA content and MMPs activity. Furthermore, increasing matrix stiffness enhances the expression of several components of signaling pathways such as Hras, RhoA, and ROCK1, thus suggesting that these pathways could be important mediators between mechanic properties of the PEG-based scaffold and the observed cell responses. The authors conclude that their platform represents a reliable 3D in vitro brain tumor system to uncover mechanisms involved in GBM progression and for screening new potential molecules in GBM pharmacotherapy. Other analogous studies compared the performance of 3D collagen scaffolds, and assessed for their capacity to screen anti-GBM drugs, with conventional 2D cultures. In the first study [121], cells in 3D scaffold exhibited greater dedifferentiation features, increased resistance to anti-GBM alkylating agents, with higher amount of GSC and upregulation of O6-methylguanine DNA methyltransferase (MGMT) than 2D cultures. Notably, tumor cells in 3D culture showed a resistance pattern to chemotherapeutic agents very similar to those observed when the same agents were tested in glioma patients. In the second paper, the authors investigated how pore size affects the expression profiles and biological functions of glioma cells cultured in collagen 3D scaffolds [122] in comparison to classical 2D cultures. Genes associated with stemness, cell cycle, apoptosis, epithelia-mesenchymal transition (EMT), migration, and invasion were upregulated in 3D collagen scaffold. Corresponding changes in the activity of diverse signaling pathways, such as proapoptotic pathways, as Wnt, Sonic Hedgehog and Notch, which regulate the observed variations in functional cellular effects, were also observed. However, the pore size of the 3D collagen-scaffold did not appear to significantly affect the gene expression or cellular functions of the glioma cells. The results of this work indicate that 3D collagen scaffolds, preserving and maintaining the major features of GBM malignity in comparison to classical 2D assays, could be regarded as a promising in vitro platform for investigations of GBM molecular mechanisms. The properties of alginate hydrogel scaffolds in supporting and influencing GSC characteristic have also been exploited. Microscale alginate hydrogel tubes were found to support GSC proliferation rate, with high cell viability and high volumetric yield for long term cultures, also preserving and maintaining stem cell features [123]. In a quite similar work, the authors [124] engineered a 3D organotypic microfluidic platform, based on hydrogel biomaterials, to study the interactions and the pathway underlying invasion and phenotype changes between GSC vascular niche and endothelial cells (ECs). The microvascular network increased GSC migration, promoted an invasive morphology, and enhanced GSC proliferation rates that maintained a stem-like phenotype. Moreover, using the CXCR4 antagonist AMD3100, the authors found that the CXCL12-CXCR4 axis is deeply involved in promoting GSC invasion in the GBM microenvironment. However, the most notable result came from the demonstration that the migration behavior and invasive morphology found in this 3D model are over-imposable and similar to an in vivo mouse model, thus elegantly validating this platform as a reliable and valuable in vitro physiological model GBM microenvironment.

#### 5.3.2. Rigid Scaffolds

Another experimental approach involves the use of rigid scaffolds for 3D cultures. Several matrices have been used and, in the following paragraph, some of these results will be discussed. Polystyrene is a widely used polymer for 3D cultures; in particular, electro spun polystyrene scaffolds coated with different isoforms of laminin were reported to drive stem plasticity with results very similar to those found with non-rigid scaffolds. When these scaffolds were coated by the laminin isoforms 411, 421, 511, and 521 and used for GSC 3D cultures, an increased expression of the GSC markers (including, for example, SOX2 and OLIG2) was found and, concurrently, an increase in clonogenicity of these cells was also observed [125]. Interestingly, in this study, an increase of integrin α6 and b4 expression was observed in 3D glioma cell culture, suggesting that such a model could be particularly suitable to study integrin-linked pathways in glioblastoma. Another group compared a customized 3D GBM culture system constituted by a polystyrene scaffold (Alvetex) with conventional 2D cultures for their capability to respond to radiation and to drugs targeting signaling pathways commonly dysregulated in GBM [126]. Among these, temozolomide and bevacizumab performed better in this 3D model than in classical 2D cultures whilst erlotinib, inactive in clinical trials for GBM [114,115], had no activity in the 3D assay, but augmented the efficacy of radiations in the 2D assay. These data suggest that this type of 3D system could be a reliable in vitro model in predicting the clinical efficacy of putative GBM anticancer agents when combined with radiation therapy. Other authors, in the attempt to study the migration potential of different of GSC, have devised a model based on the deposition of GSC in an aligned polyacrylonitrile (PAN)-derived nanofiber (NF) [127]. The study demonstrated that cells migrated on NF scaffold coated with laminin, and GSC migration is very similar to what has been observed for mesenchymal migration in vivo and, in addition, upregulation specific migration-related molecules such as galectin-3 and integrin-β1 in galectin-3 was observed, also showing within the turnover of focal adhesion molecules between single-cell and collective migration. This interesting study showed that GSC migration can be effectively reproduced in vitro by using suitable models. New, interesting hints come from the emerging carbon-based material technology, especially carbon nanotubes (CNTs), that have been widely utilized to produce scaffolds with improved mechanical strength. Due to their electrical properties, CNTs allow effective interactions with cells and modulate neuronal growth [128]. In the light of these features and among the wide array of possible applications, CNTs have also been used for the reconstruction of in vitro neuronal networks mimicking the in vivo brain connectivity, particularly to reproduce interactions between normal cortical neurons and glioma cells. In this study, cortical neurons grown on CNTs were found to develop, forming a 3D cortex-like network and to produce ECM proteins. The authors suggested that this advanced 3D model could be used to construct an ideal glioma infiltration model to map glioma cell invasion in 3D and to screen new potential drugs. In the light of recent findings dealing with interplay between GSC and normal neurons, the possibility to produce cocultures with normal reprogrammed neuronal cells of a patient and GSC derived from same patient underscores the potential wide field of application of this platform. Indeed, this system allows not only live cell imaging of GSC dissemination and infiltration into healthy brain tissues, but also could represent a valid tool for the screening of drugs that may inhibit GSC infiltration.

## 6. Organoids

**Figure 2:**
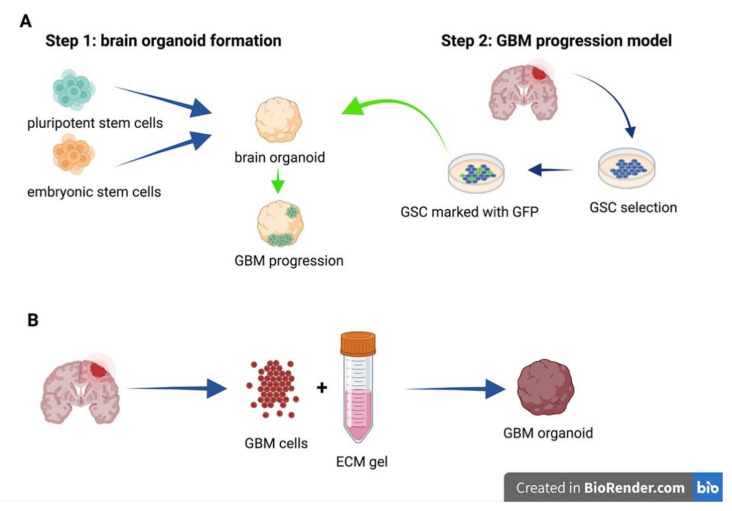
Examples of GBM organoid use in research. (**A**): GSC obtained from a patient are transfected with green fluorescent protein (GFP) and co-cultured with brain organoids, obtained from pluripotent or embryonic stem cells, to study tumor progression. (**B**): GBM organoids are obtained from GBM cells dissociated and grown in Matrigel spheres.

Despite some critical points that mainly include the identification of proper markers and the supposed heterogeneity of cell population, GSC represent today the best available model in vitro to study GBM. However, this model suffers from a severe drawback related to the absence of physiological and reciprocal interactions among GSC and other cells, such as non-tumor cells and vascular cells, belonging to the tumor microenvironment. Indeed, these two cell populations entail a reciprocal interplay with GSC in the tumor niche. Non-tumor cells and vascular cells feed and support GSC growth either by releasing soluble molecules and by shaping the ECM; GSC, in turn, produce angiogenic factors, thus influencing the differentiation and pro-tumorigenic activity of surrounding vascular perycites [47]. In the attempt to partially fill this gap, several groups have developed and proposed the organoid model (Figure 2). Organoids could fulfil the promises of an ideal in vitro model: they resemble the in vivo architecture of the tissue of origin and at least theoretically recapitulate cell growth, self-organization, and differentiation, thus mimicking the cell heterogeneity found in the tumor microenvironment [48]. Neural organoids were described in a study that first reported the generation of reconstituted in vitro neural tissue, derived from human pluripotent stem cells (hiPSCs), that retain the original spatial organization of the developing cortex [49]. In recent years, the field of organoids has gained an enormous popularity and has received a peculiar attention from researchers interested in developing and improving this type of 3D model either in brain physiology and brain physiopathology, such as brain cancer [50,51,52,53]. These reviews inspired other scientists who dare to translate this innovative approach into the field of in vitro GBM models aimed at modeling primary human GBM ex vivo and at establishing reliable and suitable high-throughput drug screening. In a recent paper, the authors retro-engineered patient-derived glioma stem cells and human embryonic stem cell (hESC) to form glioma cerebral organoids termed GLICO [54]. In this model, GSC tumors invade and proliferate within the organoids, thus self-assembling a tumoral mass and environment that resemble very closely the original patient GBM. In addition, these cerebral organoid tumors, during their formation, develop an interconnected network of microtubes that allow the invasion of normal host tissue. Notably, this last feature is a remarkable advantage over other models cited in this paper, because it exquisitely fits with the most recent finding in the interplay between GBM tumor cells and normal brain tissue. In an analogous study, a long-term 3D organoid culture that supports tumor growth and differentiation was obtained directly from multiple regions of GBM specimens with the purpose to comprise a mixture of heterogeneous cell population [113]. This model of tumor organoids is unique in its architecture being characterized by an outer region of rapidly dividing cells that surround an inner hypoxic core of primarily non-stem cells and diffuse quiescent GSC. The functional validation of the reliability of this model was demonstrated by the finding that orthotopic transplantations of these organoids led to the formation of tumors with better histological and invasiveness pattern compared with parental patient-derived neurosphere cultures. In a similar approach, other authors leveraged the CRISPR/Cas9 genome editing technology applied to GBM cell lines or patient-derived cells grown as neurospheres to assemble an organoid system that homes very invasive transformed cells [55]. These organoid-derived putative tumor cells, whose gene expression pattern resembles a mesenchymal subtype human GBM, once transplanted into human cerebral organoids, display invasive tumor-like structures, thus indicating the reliability of this platform as a promising tool in elucidating molecular mechanisms involved in GBM malignancy. A new type of organoid was set up and studied as a reliable model to recapitulate the intrinsic inter- and intra-tumoral heterogeneity of GBM in a very recent and comprehensive paper [129]. The authors, using sophisticated, state of the art histological, transcriptomic, genomic, and single cell analysis approaches, generated and bio-banked patient-derived glioblastoma organoids (GBOs) that not only, in vitro, closely mirror gene features of their parental tumors, but in addition, they display malignant infiltration and the ability to form tumoral masses when transplanted in in vivo animal models. The major advantages of this model, compared to other previous published organoid-like models generated from dissociated tumor cells of epithelial origin and propagated over matrigel, are represented by the finding that these GBOs retain original cell–cell interactions and are cultured in EGF/bFGF free medium without ECM components. Therefore, this novel approach represents an important leap forward improving and refining patient-specific treatment strategies in the context of personalized medicine and translational GBM research. Despite these encouraging results, but taking into account that organoids are still in their early infancy, this approach suffers from several drawbacks; similarly to other in vitro models, cerebral organoids lack some essential cell types that play an important role in tumor malignity. GBM preferentially infiltrate brain tissues by moving along existing blood vessels and, therefore, the absence of endothelial cells is a severe pitfall; coculture with endothelial cells or mesenchymal progenitors could solve this issue. On the contrary, brain organoids may contain cells deriving from tissues non belonging to or not directly present in the GBM microenvironment [55]. Poor reproducibility is another critical issue with highly variable results, because brain organoid culture, in general, requires specific skills and takes a long time (usually months of culture) before being ready for morphological and functional assays. Strictly related to this point, the culture conditions and medium composition are not yet standardized, thus requiring further studies. Additionally, the proportion of different cell types required in the initial phase of assembling the 3D model varies in the different organoid models and cannot be standardized. In conclusion, due to the extreme complexity of this heterogeneous model, standardization of culture conditions is a daunting task that must be accomplished only by dedicated multidisciplinary efforts from the GBM community.

### Organotypic Slices

Another approach similar to organoids that could be useful to study GSC–host interactions is represented by organotypic slice cultures [56]. Several studies have used human postnatal brain slices maintained in serum or slices dissected from brain rodents previously treated with chemical agents or injected with GSC or GBM cell lines to induce brain tumors. Although the use of these latter models is mainly restricted to invasiveness and dissemination studies, they are gaining widespread acceptance in GBM experiments, because they mimic very closely a physiological environment. In an elegant paper [57], human GSC were microinjected into specific anatomical sites of whole adult brain coronal organotypic slices kept viable in a serum-free basal medium for several weeks. Different responses of engrafted GSC to diverse microenvironments in the brain tissue were observed. When GSC were injected in slices derived from specific brain areas, such as subependymal zone, they responded to endothelial niche signals and demonstrated a decreased viability and survival upon treatment with the antimitotic drug temozolomide. This approach could therefore constitute a valid proof of principle in implementing a flexible and versatile experimental tool to study GSC–host brain interactions and to get further insights into the mechanisms that drives GSC dissemination and invasiveness. Another study has developed a quantitative method to assess glioma invasiveness, providing an important tool in developing pharmacological and genetic assays aimed at screening putative inhibitors of glioma invasion [58]. The authors seeded patient-derived GFP-labeled GBM cell lines into organotypic mouse brain slices and then monitored the invasion of these cells into the brain tissue by using confocal microscopy. Among the subpopulations of GBM cells, only the mesenchymal GBM subtype displayed a strong invasive capacity; this feature was ascribed to a difference in the transcriptomic profile, characterized by enrichment of several ECM-related components, of these cells compared to a less invasive subtype. The authors, using pharmacological and genomic tools, identified interferon regulatory factor 3 (IRF3) as the transcriptional repressor of ECM-related pro-invasive genes and conversely the serine/threonine protein kinase Casein Kinase II (CK2) as pro-invasive agents by negatively regulating IRF3 activation. As expected, CK2 inhibition results in downregulation of these ‘pro-invasive’ ECM genes whilst genetic silencing of IRF3 increased glioma invasiveness. This intriguing finding implicates the notion that increasing IRF3 expression, associated with a concomitant blockade of CK2 activity, could be exploited as innovative approach to reduce glioma invasiveness. In addition, future experiments using this 3D model could provide further insights into the hypothesis, already supported by previous works [59], that glioma invasiveness and glioma proliferation are two key processes sustained and modulated by different signaling pathways. In another report, stem cell-like containing spheroid (GSS) cultures, directly obtained from patients, were implanted into in vitro rat brain slice cultures kept in a stem cell medium and also in vivo into brains of immuno-compromised mice [60]. When invasion, proliferation, and expression of stem cell markers of GSS in the two models was evaluated by confocal time-lapse microscopy and immunohistochemistry, the authors found that proliferation and expression of stem cell markers was different between implanted and free-floating spheroids. However, the demonstration that the in vitro GSS invasiveness closely resembles the in vivo invasiveness led the authors to conclude that their system could be regarded as a suitable in vitro model to study GBM dissemination and invasiveness. In another study [61], fluorescently labeled tumor spheroids were implanted into adult organotypic brain slices, but cell invasion was assessed by measuring the average cumulative sprout length per spheroid. The authors tested the effect of small molecule inhibitors in normal slices or in slices derived from genetically engineered mice that lack cell surface proteins and found that, under both experimental conditions, tumor cell invasion was severely impaired, thus proving the reliability and validity of this systems to investigate the effects of manipulations of environmental parameters concurring to GBM cells invasiveness. Another study, carried out in a similar model, focused on the migratory behavior of tumor cells in response to anticancer drugs [62]. The slices were directly obtained from human GBM tissue during surgical resection and then the migration of retrovirally labeled cells was monitored by time-lapse laser scanning confocal microscopy in conjunction with cell tracking. The authors validate this platform as a reliable in vitro tool to uncover genetic factors associated with migratory behavior in human GBM and to screen drugs aimed at reducing the GBM cell migration in the context of personalized neuro-oncologic therapy. GBM slices cultures have been also used as model to investigate the effects of irradiation or treatment with the brain antitumor drug temozolomide [63]. Temozolomide treatments, with significant differences among individual tumors, resulted in caspase 3- mediated cell death, inhibition of proliferation and cell loss, and irradiation-induced DNA double-strand breaks. This study proposes another application of GBM slices model as a tool to uncover factors involved in response of individual tumors to specific therapies and to study the molecular mechanisms underlying tumor resistance to drug treatments.

## 7. Bioprinted Chip Systems

**Figure 3:**
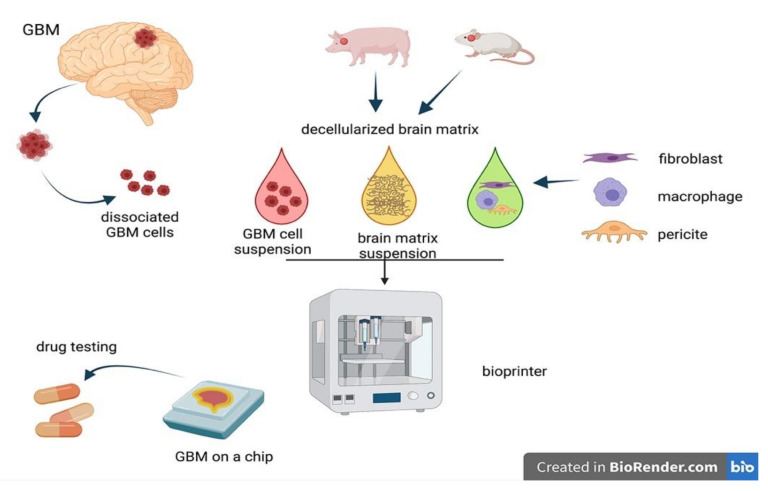
The bio-printing technique is used to create GBM on a chip. GBM cells obtained from a patient are stratified with decellularized brain matrix and other human cells present in the tumor niche. The GBM on a chip may then be used for drug testing.

An ideal in vitro tumor model should reproduce the interactions among tumor cells and other non-tumor components, possibly in an ECM-like supporting scaffold, in order to replicate the tumor niche microenvironment. A recent in vitro model, derived from approaches in regenerative medicine, that holds the promise to partially satisfy these requirements, is represented by 3D bioprinting (Figure 3). The principle of bioprinting is quite simple and straightforward, being adapted from classic and well-established 3D material printing procedures and using a slight modification of 3D printing machines. Basically, 3D bioprinting is a sort of additive manufacturing that assembles layer-by-layer biological constructs using a spatially and timely addition of viable cells, biomaterials, and biological molecules to produce scaffolds characterized by a specific and desired microarchitecture [64]. Currently, there are several different static and dynamic methods of layering the components of these 3D structures over a wide choice of supporting natural and synthetic polymeric materials, already discussed in the previous scaffold section, that form the scaffold structures. Bioprinting techniques display some benefits over classical scaffolded or unscaffolded 3D models; the role and functional effects of vascular elements in tumor cells survival can be investigated in microvascular-like structures obtained by positioning endothelial cells within the 3D structures as a pre-vascularization step prior to subsequent layering of other cell types. In addition, a correct vascularization assures a homogeneous influx of oxygen, nutrients, and efflux of metabolites and byproducts, thus preventing gradient-dependent cell death and core necrosis. Another advantage of 3D bio-printed models relies on the possibility to provide homogeneous spatial cell distribution, preventing the formation of cell density gradients from the surface toward the inner part of the scaffolds, a problem that accompanies classical cell seeding in solid scaffold structures. Although, currently, there are few examples of the application of this innovative approach to cancer studies, a study has recently and successfully reported the implementation of a bio-printed 3D GBM model that could pave the way for future studies in this field [65]. Indeed, with the constant aim of determining the most appropriate cancer treatment for individual patients, these authors demonstrated that reconstituted patient-derived GBM cells and vascular endothelial cells, layered by bioprinting on decellularized ECM compartmentalized stroma-like structure, recapitulate the functional biochemical features of the original tumors. In addition, this GBM-on-a-chip model could be used to identify the combined effect of the resistances of tumor cells to anticancer agents with concurrent chemoradiation, thus assessing the best therapeutic mixed scheme or drug combination associated with superior efficacy and minor toxicity. This patient-specific tumor-on-a-chip model might be instrumental in the screening of effective treatments for GBM patients who do not respond to standard first-line guidelines treatment. In another similar study [65], the authors set up a 3D bio-printed GSC model based on porous gelatin/alginate/fibrinogen hydrogel scaffold to partially mimic ECM. Bio-printed GSC retained their cancer stemness features, showed differentiation, and expressed tumor angiogenesis biomarkers over long-term culture periods. Finally, the 3D printed tumor model, compared to the analogous 2D model, was more resistant to temozolomide, thus confirming and extending the reliability of this assay as a promising tool to study drug efficacy and toxicity in vitro. In addition, 3D bioprinting and scaffold-free 3D tissue cultures have been used to investigate the invasion of GBM cells into mouse neural progenitor cell-derived spheroids [66]. In this study, the authors found that both approaches could reliably be adopted to study the invasion of glioma cells into neural-like tissue by 3D confocal microscopy and by cell tracking dyes both in fixed samples and in real-time conditions.

### The Unsolved Issue of Blood–Brain Tumor Barrier

Finally, the role of the blood–brain tumor barrier (BBTB) in a GBM model should be considered. Normally, the blood the brain barrier (BBB) is mainly composed of brain endothelial cells that line the inside part of vessels; however, the most important functional mechanism is represented by the tight crosstalk between endothelial cells and brain cells, such as astrocytes and pericytes, that tightly regulate the passage of molecules towards the brain. While, in low-grade gliomas, the normal architecture and function of the BBTB still resemble the normal BBB, in high-grade gliomas, major alterations are observed, with a leaky BBTB resulting in a loss of the normal vascular function [67]. Therefore, BBTB appears to be a key element in determining GBM response to drugs and to immune system attacks. In the attempt to study the role of BBTB in GBM, several human in vitro BBTB models, consisting of a tertiary system composed by co-culture of human endothelial cell, pericytes, and glioma cells have been implemented. Although these current systems bear some problems and limitations, they pose the basis for the future development of suitable and reliable BBTB in vitro platforms aimed at refining and improving the targeting of drugs toward GBM malignity [68].

## 8. Perspectives and Trends

In conclusion, there is no single “best experimental model” to study GBM, even though 3D models appear to be useful to study complex multicellular systems and the reciprocal cell–cell interactions among tumor and non-tumor cells; in addition, 3D models provide realistic modeling of brain-like structures with partially retained original architecture, allowing studies on ECM and attachment factors in soft or rigid scaffolds of different matrices. Indeed, some labs use a combination of both 2D and 3D cultures, sometimes called “2.5D cultures,” or also “3D-like models”, thus leveraging the advantages of 2D assays implemented into a variety of 3D architectures. Another important issue to be considered is the apparent minor drug sensitivity of 3D models compared to 2D cultures. Although this discrepancy has not been reported by all studies and therefore is still a subject of an open debate, it is conceivable and widely accepted that this effect is due to an intrinsic difficulty of drugs in accessing the inner part of the 3D systems. However, this effect appears to be strictly model type-dependent and several parameters, like scaffold stiffness and porosity, together with cell density and distribution, affect this issue and should be carefully considered when setting up a 3D GBM model. In addition, as in other cancer models [69], the combination of 3D cultures and dynamic milli-fluidic systems may help to better mimic the drug distribution and absorption observed in tissues in vivo. The main limitations of existing 3D methods also include poor scalability, repeatability, sensitivity, feasibility, and compatibility with high-throughput screening (HTS) assays and instruments. Analysis of cellular response, such as cell viability, proliferation, migration, and invasiveness to screen drug efficacy and toxicity requires the routine use of commercially available quick assays whose translation, except in few cases, has not been tested and validated in 3D cell models. Although the number of papers describing 3D models in the literature is increasing at a very rapid pace, no 3D methods have completely replaced classical 2D culturing, because data on drug interactions, cell differentiation, modifications of signaling pathways, and long-term viability are still limited. Another important limitation of classical 3D model deals with the failure to recapitulate the complex dynamics among all the different cell variety occurring in the tumor niche. In the attempt to overcome this flaw, many research teams are concentrating their efforts in developing “tumor-on-chip” systems [130]. These systems combine the notable advantages of microfluidic platform such as great scalability, the possibility to use very low amounts of cells, and the ability to use different cell types in direct contact or separated by synthetic or natural interfaces, to study interactions between ECM-like substrates and cells on a microscale dimension, to implement tissue-like models for personalized medicine and finally to screen novel agents for their anticancer activity. It is expected that, in the short future, other technologies related to tumor-on-chip, such as 3D printing and bioprinting, and high throughput multi-omics coupled to computational micro-fluido-dynamic modeling will greatly contribute to the large diffusion of these devices. The intra-heterogeneity and inter-heterogeneity of glioma cell populations represent an impressive and sisyphean task in implementing reliable in vitro 3D models. This consideration is especially true and appears to be crucial when dealing with the crosstalk among cancer cells and other non-tumor cells present in the tumor niche. This issue can be addressed by analyzing cells subtypes in vitro using innovative single cell multi-omics techniques; indeed, several papers have reported single cells transcriptomic, genomic, and epigenomic analyses [131] whilst, despite some recent findings [132], single cell proteomic analysis is lagging behind in its routine implementation. However, these approaches still suffer of a major critical drawback: the relative proportion of the different GBM cell subtypes and other non-tumor cells originally found in situ in tumor mass is lost and, therefore, the reciprocal functional interactions among these cells still remain a grey area. Hence, in the attempt to fill this gap, a research team took single cell analysis a step further by developing the spatial transcriptomic slide-seq technique [133]. This approach, still very new and, so far, applied to the study of cellular heterogeneity in normal human brain slices, could be in the next future successively tailored and implemented in human GBM specimens to derive the spatial localization of cellular diversity in GBM, together with the wide variety of non-tumor cells that form the tumor microenvironment. This information could be instrumental in planning the relative proportion in 3D models. Moreover, precisely in this regard, recent findings have shown mechanisms of GBM dissemination and tumorigenicity that could deeply impact the current pharmacological approach to GBM therapy. Three research teams have demonstrated independently that GBM tumor cells can also communicate with surrounding healthy neurons via the formation of a neural network characterized by active synapses [83,97,134].

Instead of representing a disincentive and deterrent in this field, these recent findings should sound as a pressing plea to biomedical scientists for spurring new research studies, aimed not only at improving and refining already existing models, but also at implementing innovative 3D in vitro systems. Nowadays, there is no ideal 3D model that could satisfy all the unanswered questions posed by GBM related issues, but improvements in automation and cost reductions could give a boost in this context.

## Figures and Tables

**Table 1:** Summary of the advantages and disadvantages of all the techniques cited in the text.

Model/Technique	Strengths	Weaknesses	References
Glioma cell lines grown in 2D	Homogeneity of cell populations.	Genotypic and phenotypic variations.	[13,14,15]
	Commercially available. Suitable for high-throughput drug screening.	Very different growth conditions reported in the literature.	
		Do not closely resemble GBM.	
Glioblastoma stem cells (GSC) 2D–3D cultures	Share features of GBM (resistant to therapeutic treatments, high invasiveness). Grown as adherent cells or neutrospheres.	Must be isolated from fresh human samples. Extensive characterization required. Composed by non-homogenous cell populations. Spheres environment could limit stem cell divisions.	[2,14,16,17,18,19,20,21,22,23,24,25,26,27,28,29,30,31]
Glioma cell lines grown in 3D	Enhanced invasiveness. Increased integrin expression. Expression of stemness markers.	Not well characterized middle ground between cell lines and GSC.	[32,33,34]
Microtubes	Allow imaging analysis. Possibility to study intercellular communication and niche formation.	Effect of cell spatial arrangement and identification of structural markers critical.	[35,36,37,38,39,40,41,42]
hiPSC	Fresh GBM specimen not required. Can be produced in lab by genetic manipulations.	Genetic manipulations may not reflect genotype of GSC from human samples. Technically complex to obtain.	[43,44,45,46]
Organoids	Resemble the cell heterogeneity of the tumor microenvironment in vivo. Suitable to study the niche microenvironment. Suitable to study cancer cell invasion. Cell populations can be genetically manipulated.	Organoids composition may vary between different experiments. No standard protocol reported in the literature. Results not easily reproducible.	[47,48,49,50,51,52,53,54,55]
Organotypic slice cultures	Useful to study infiltration processes.	Mouse brain slices required.	[56,57,58,59,60,61,62,63]
Bio printed chip systems	Possibility to build 3D microstructures of various cell patterning in microfluidic devices.	Critical choice of supporting scaffolds composition and bio ink printability.	[64,65,66,67,68,69]
